# Supplementary material for: Pelage variation and morphometrics of closely related Callithrix marmoset species and their hybrids
Source: BMC Ecol Evol. 2024 Sep 20;24:122. doi: 10.1186/s12862-024-02305-3 (PMC11414090; doi:10.1186/s12862-024-02305-3)
Supplement: Supplementary file 1 — Additional file 1. Pictures showing labeled facial regions used for phenotypic identification of sampled hybrids. Callithrix species were distinguished by: color of the lateral sides of the face; coloration in the frontal and back portions of the vertex; coloration, shape, and volume of the auricular tufts; presence/absence of a white forehead marking; coloration of the orbital region; and coloration of the menton region. [file 12862_2024_2305_MOESM1_ESM.pdf]

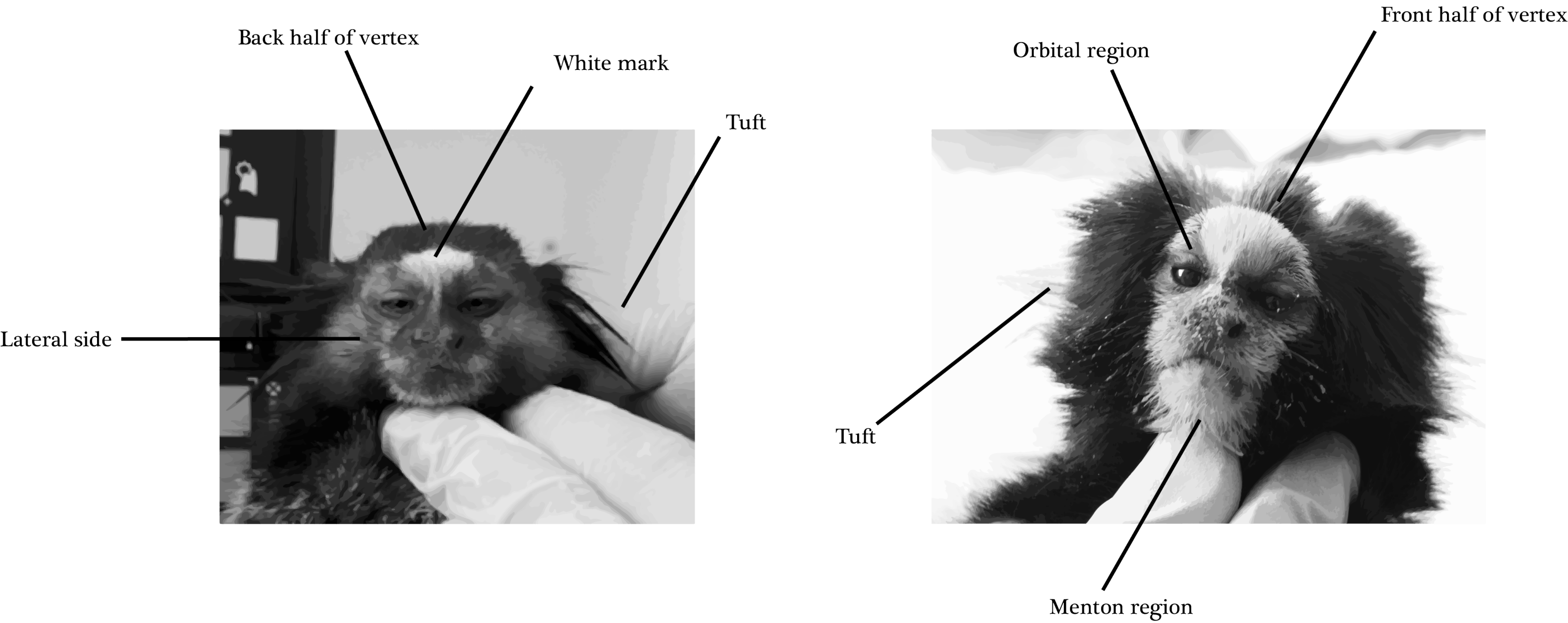
Back half of vertex
White mark
Tuft
Lateral side
Orbital region
Front half of vertex
Tuft
Menton region
