# Supplementary figures and images for: Pelage variation and morphometrics of closely related Callithrix marmoset species and their hybrids
Source: BMC Ecol Evol. 2024 Sep 20;24:122. doi: 10.1186/s12862-024-02305-3 (PMC11414090; doi:10.1186/s12862-024-02305-3)

Body

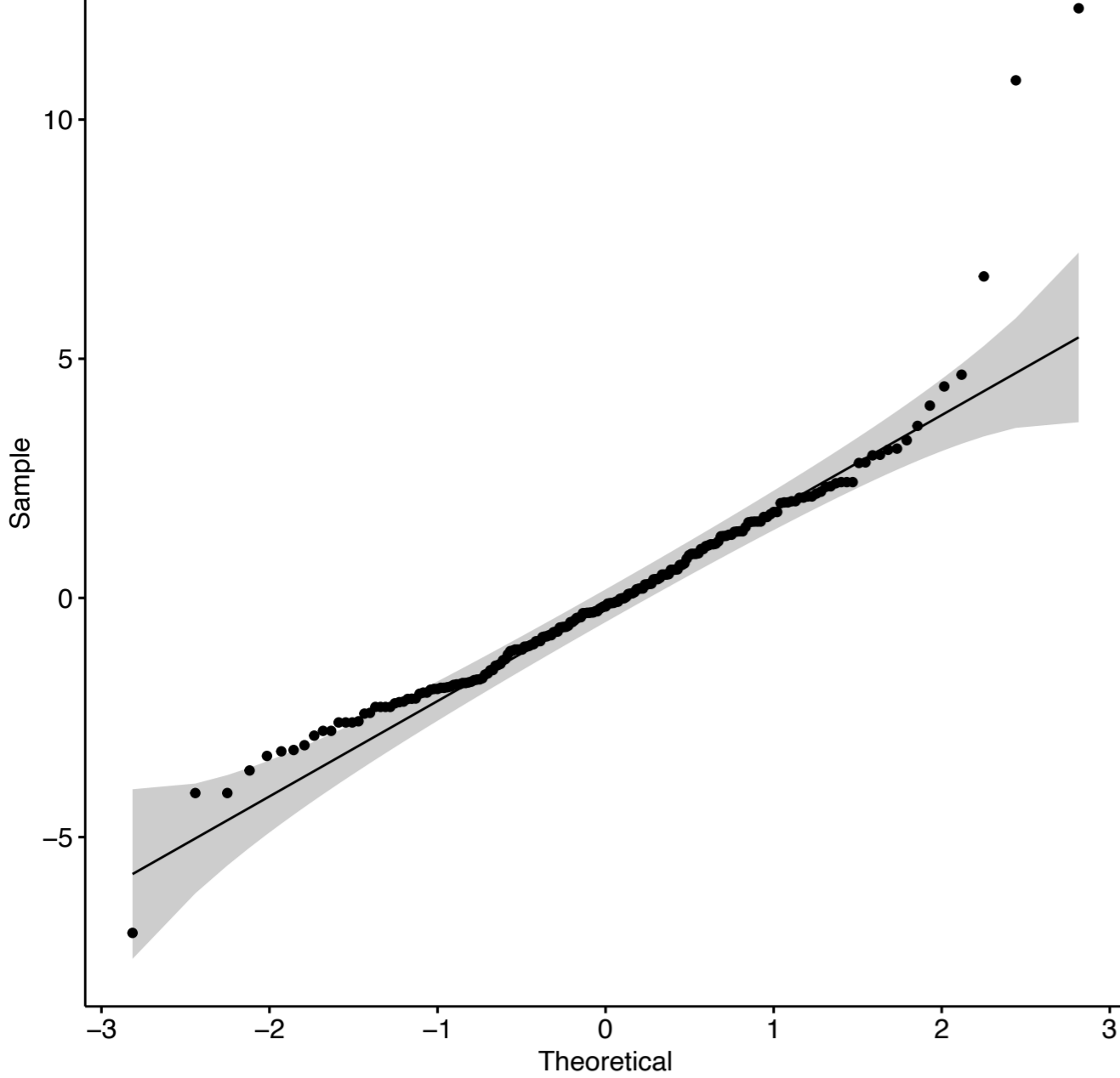

Femur

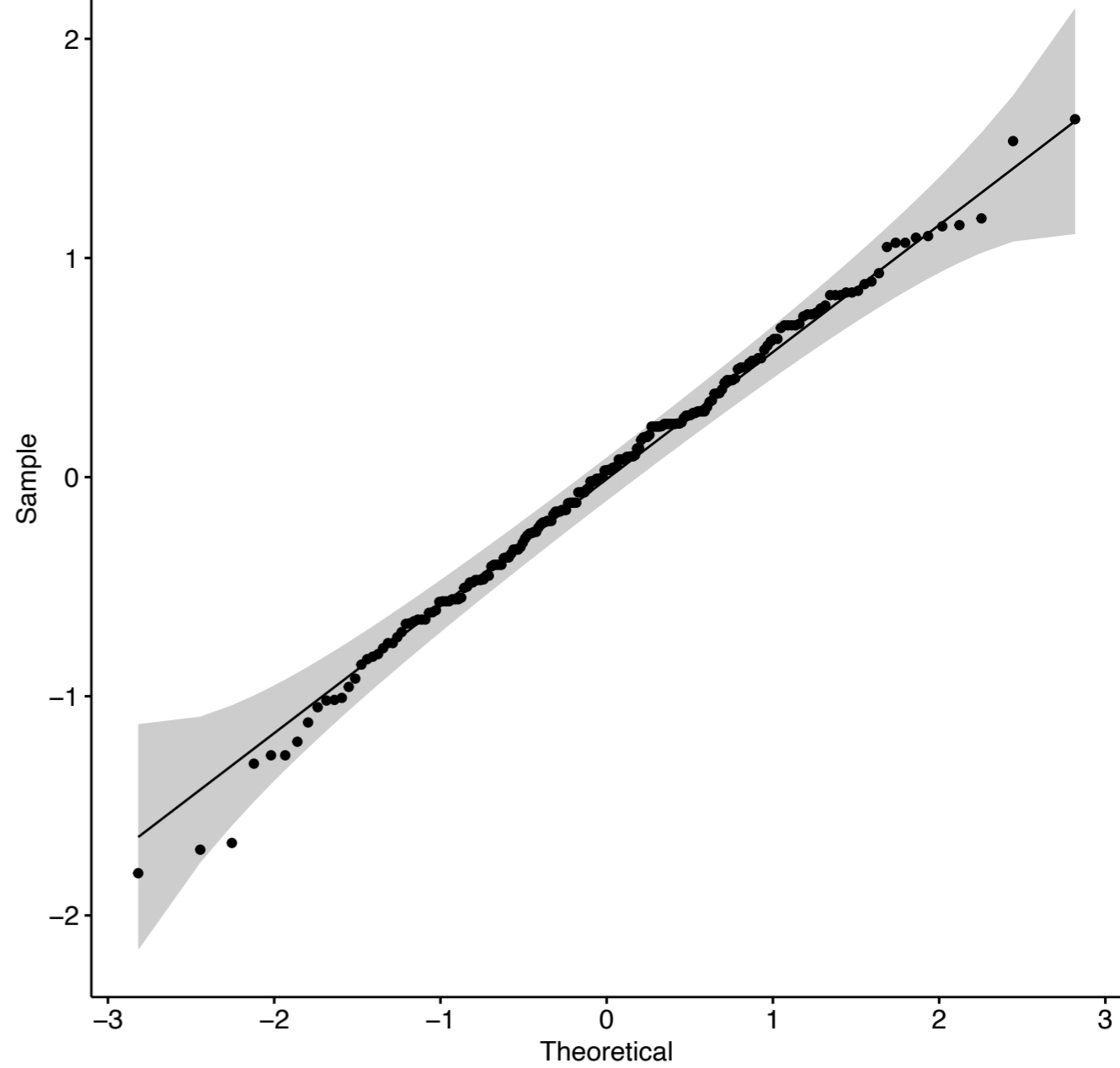

Forearm

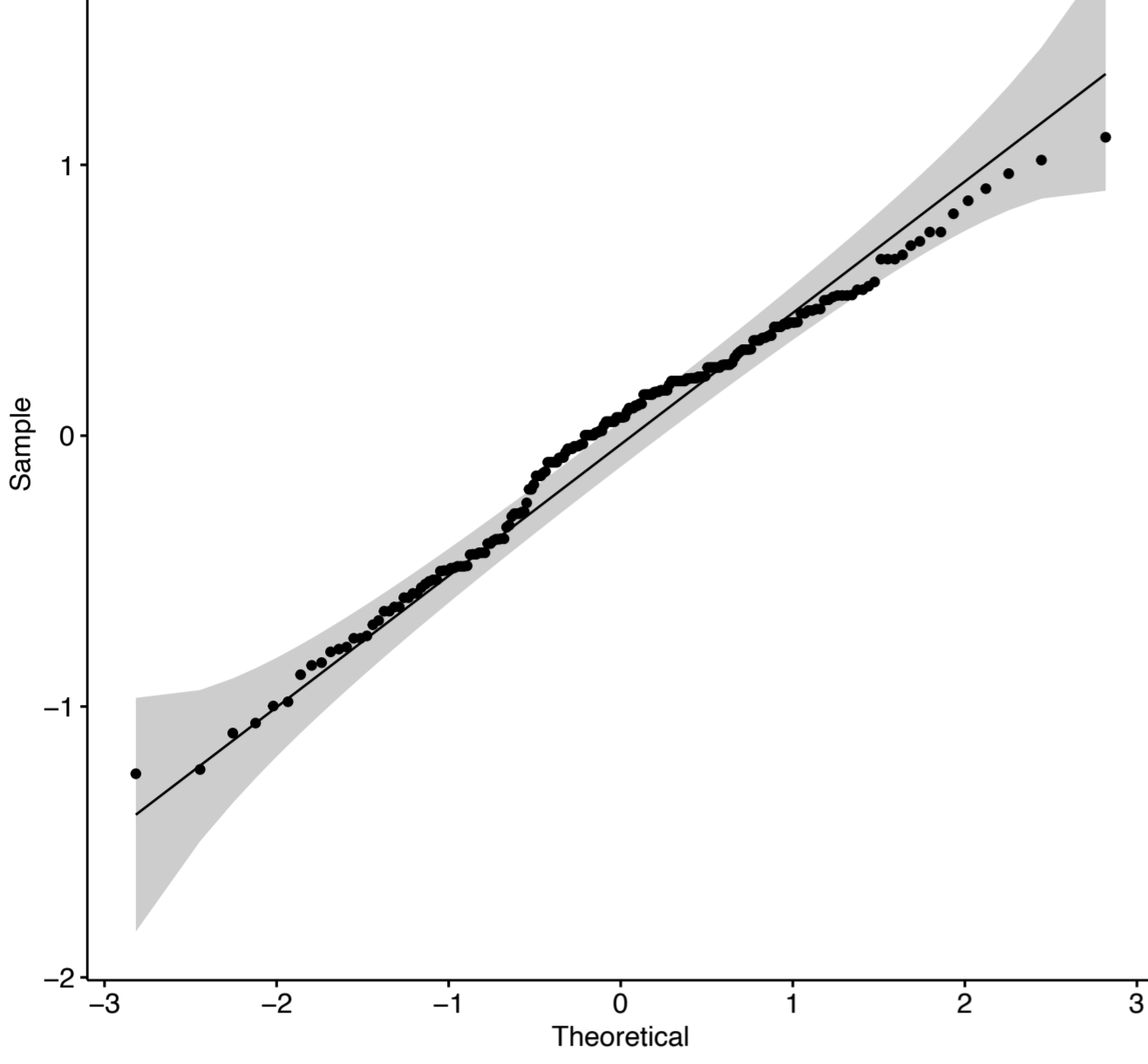

Foot

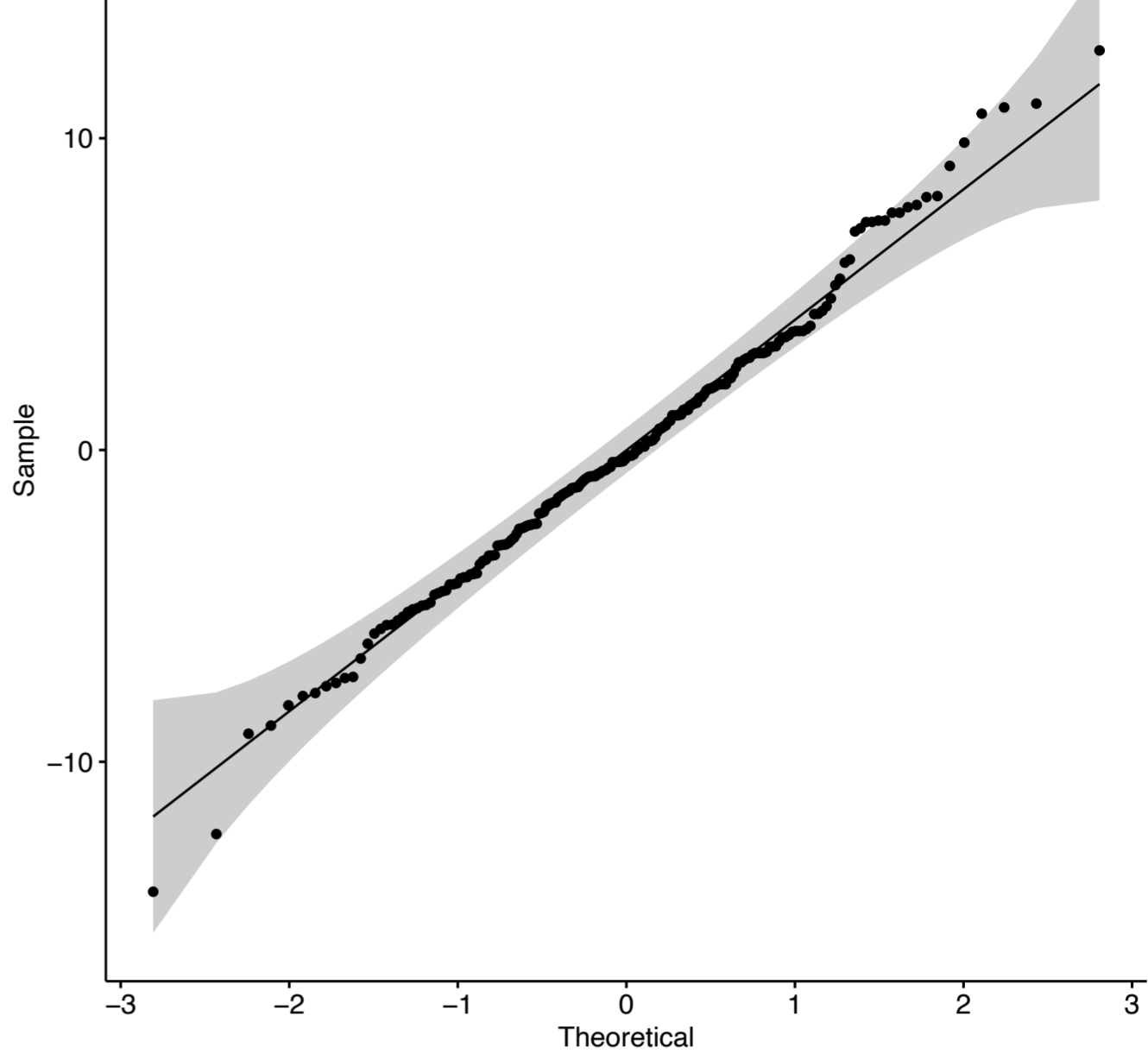

FO

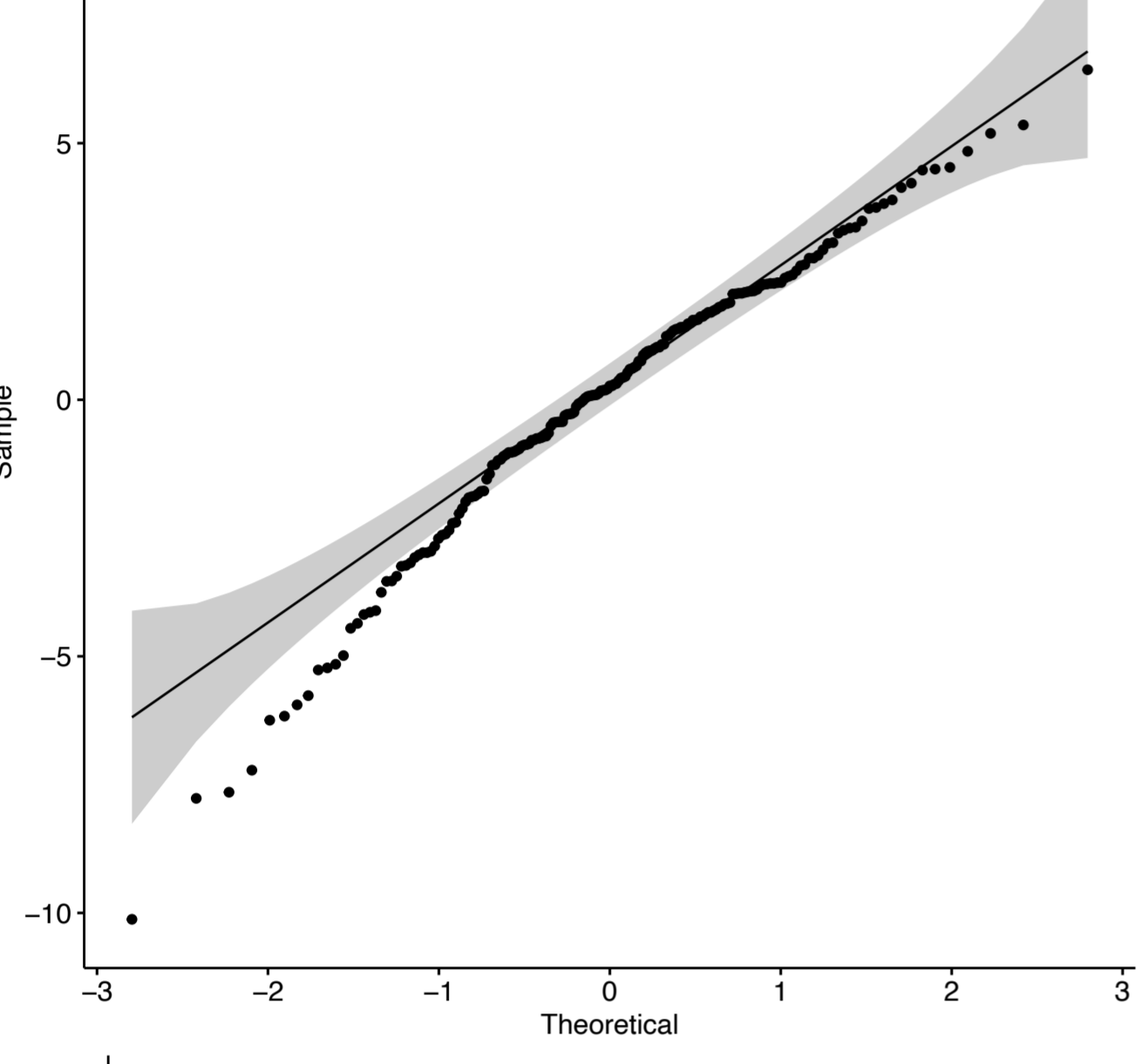

Hand

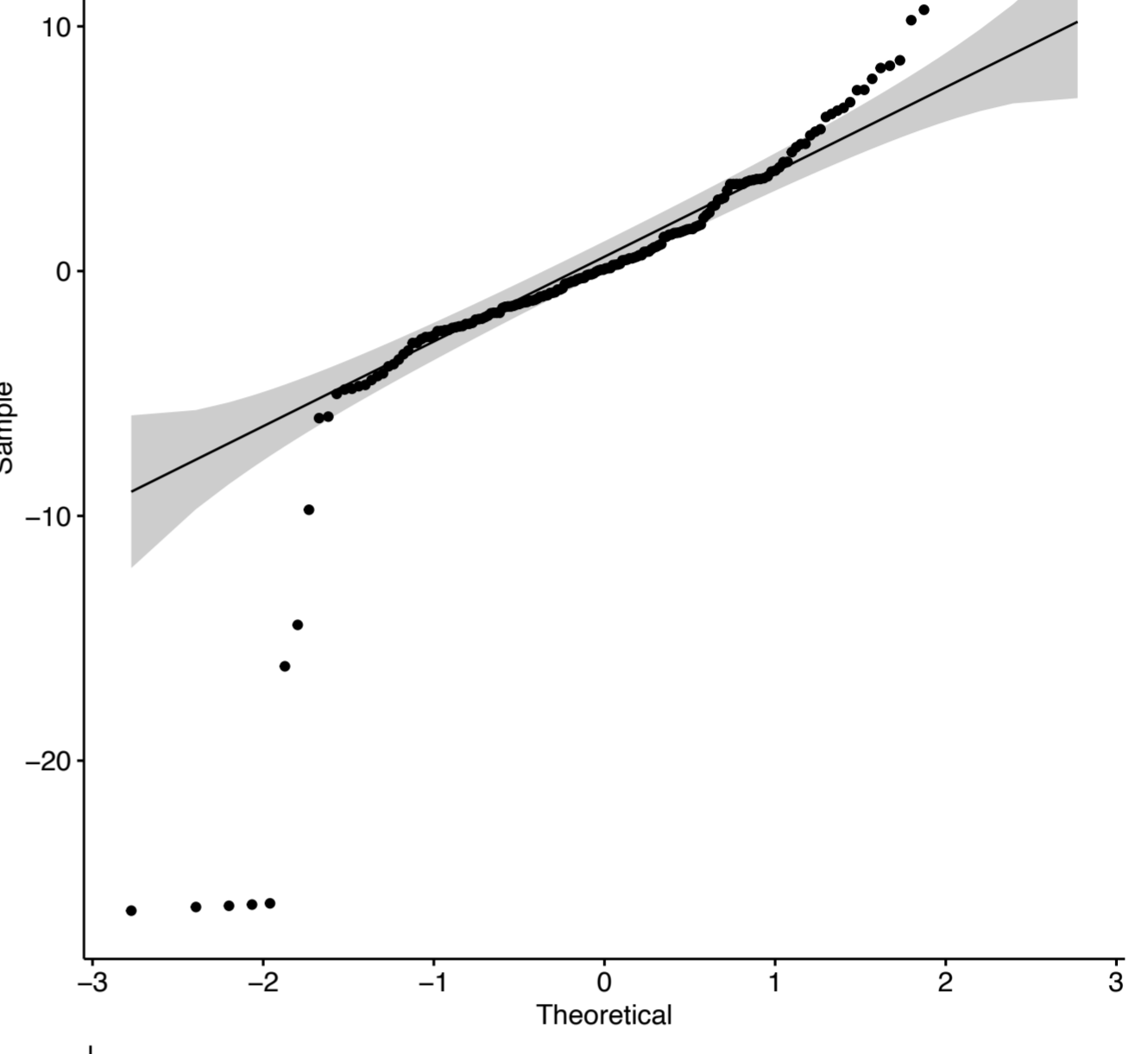

Humerus

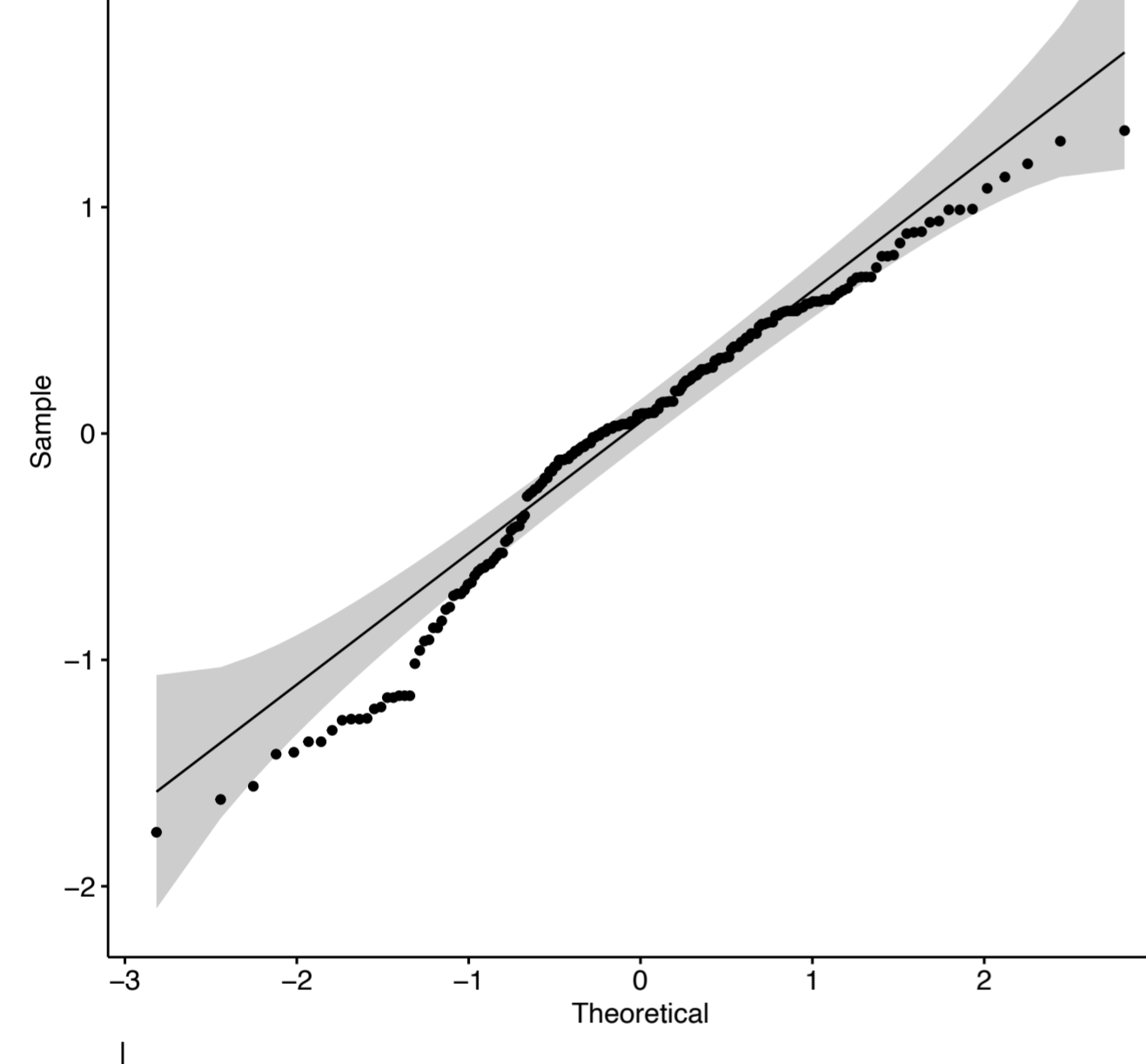

IC

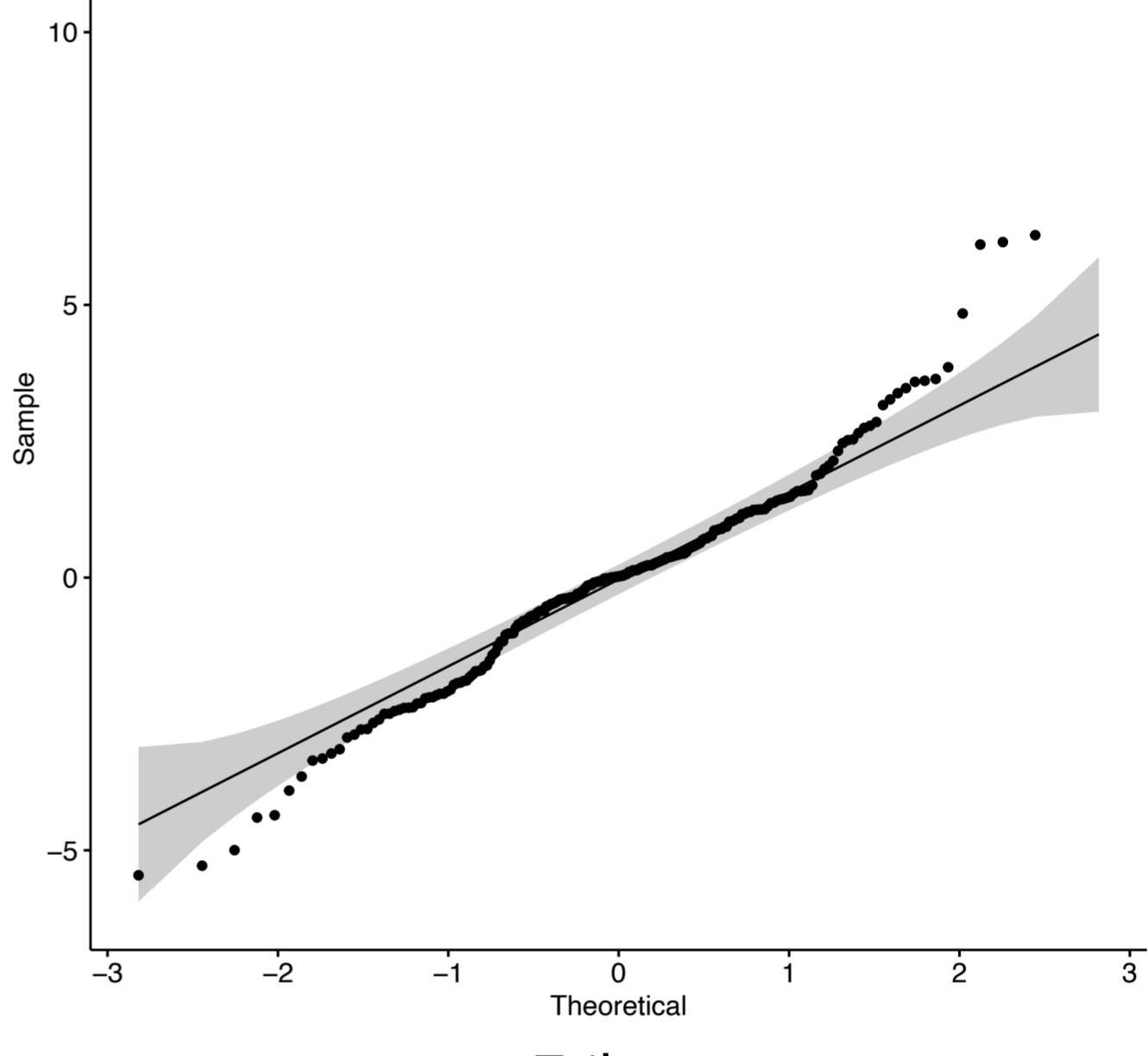

Jaw

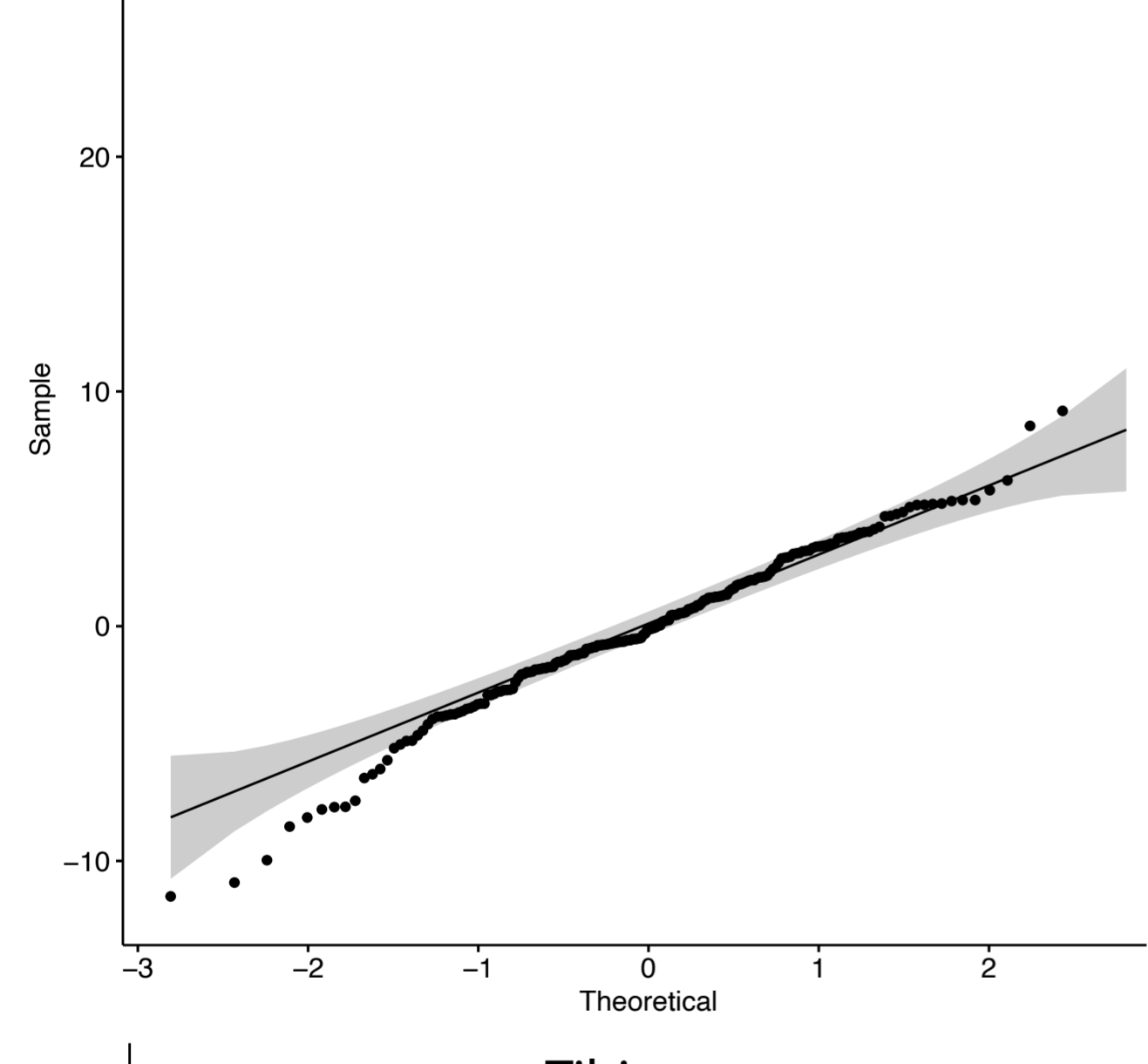

Tail

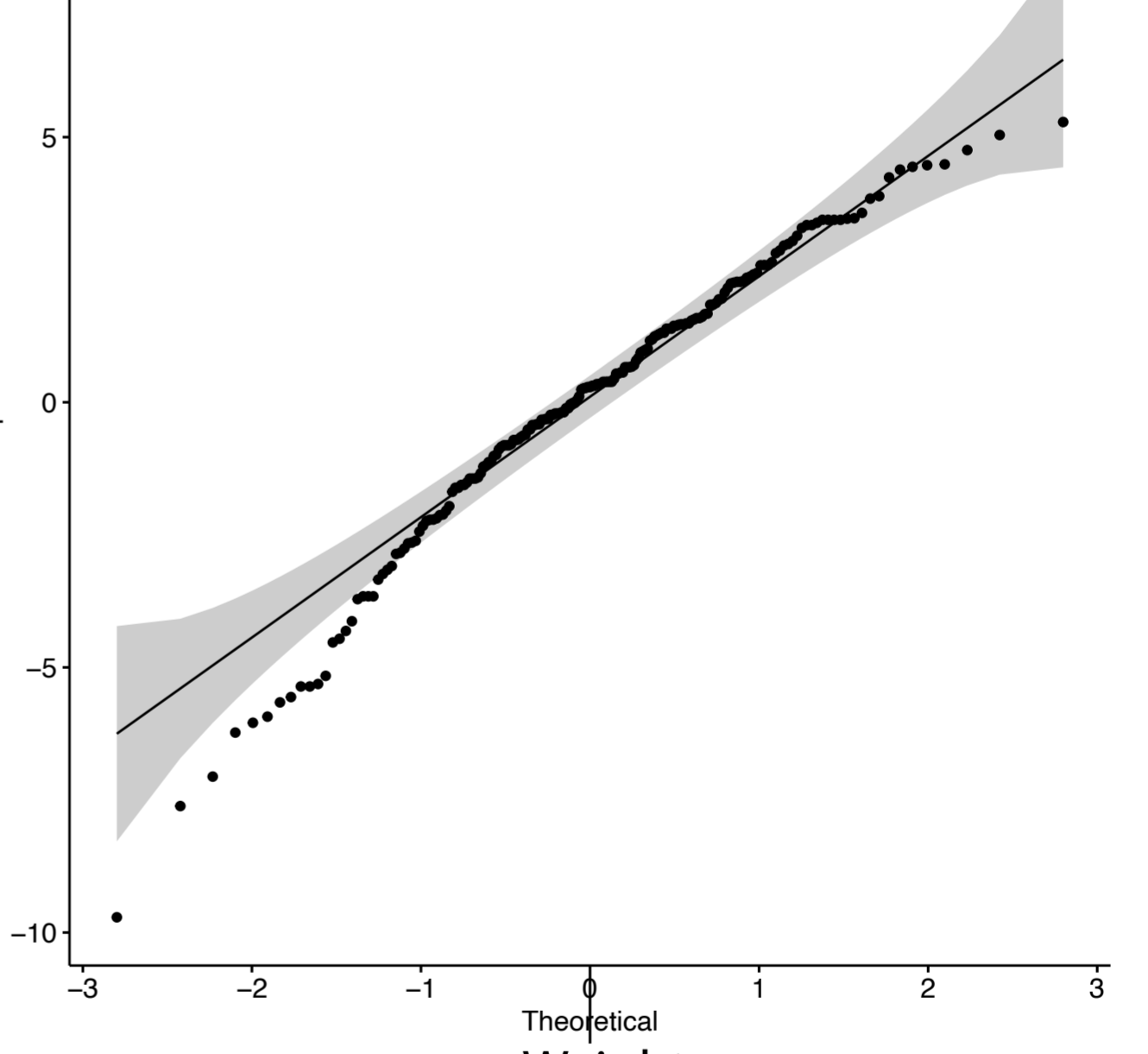

Tibia

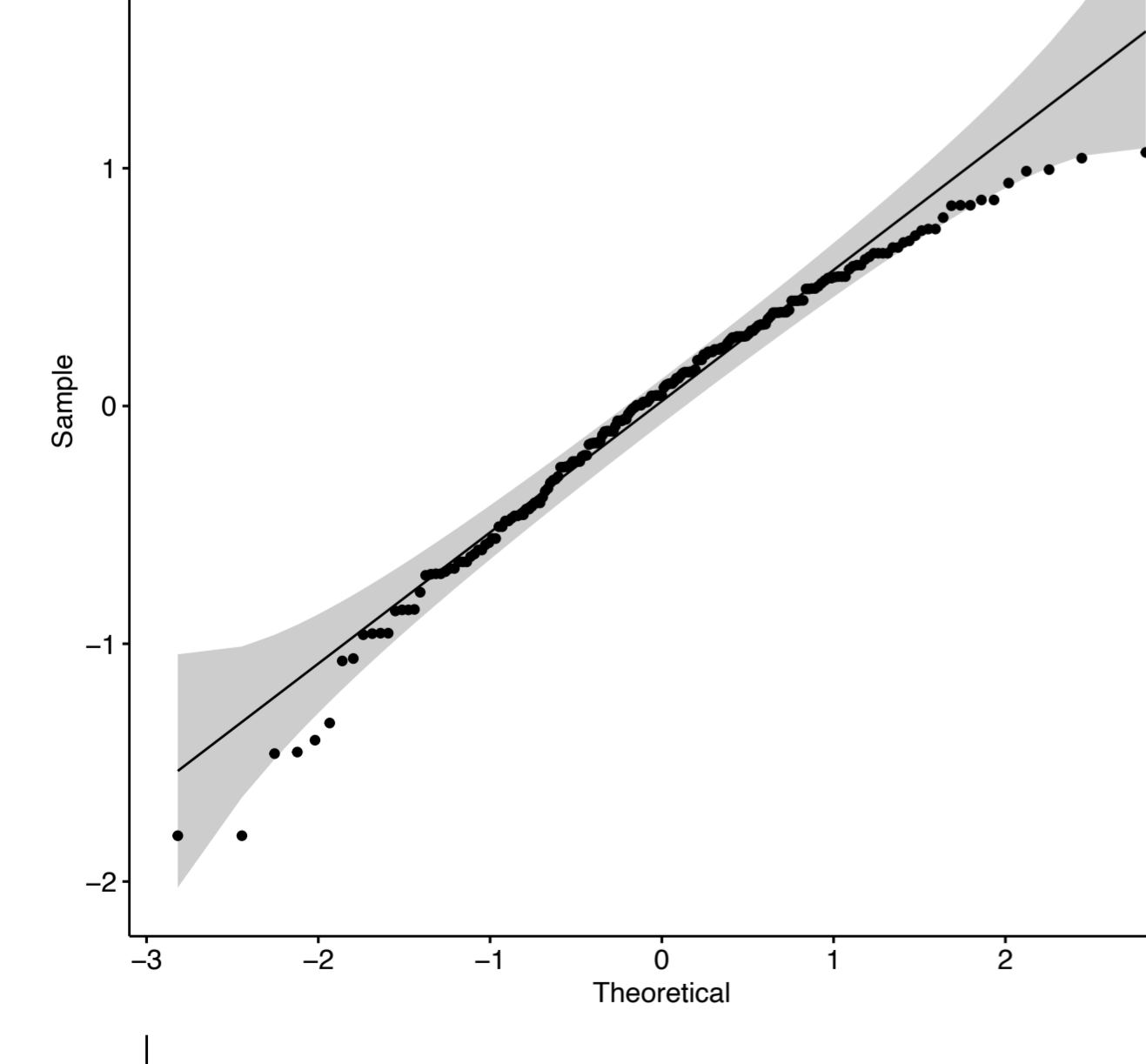

Weight

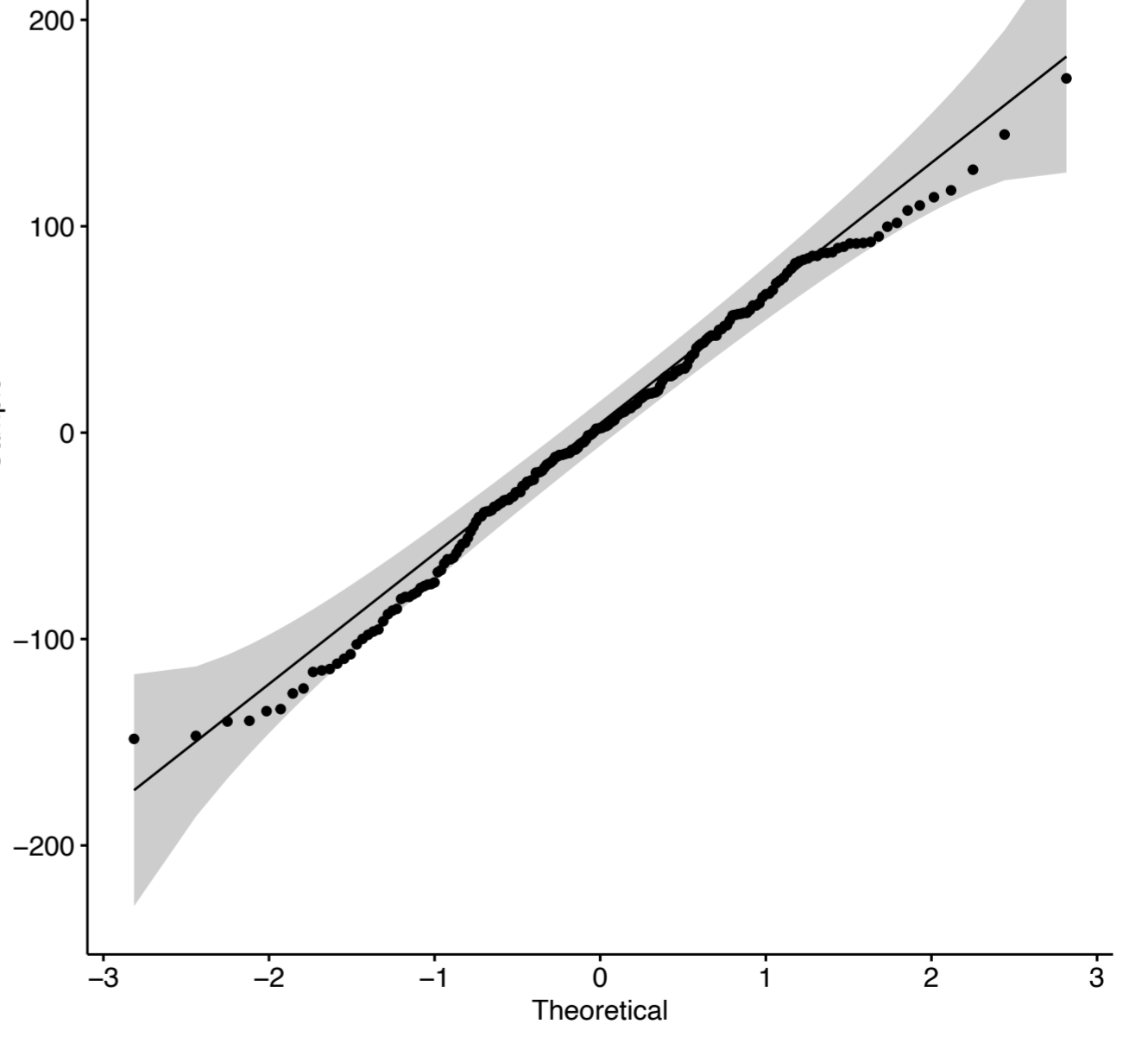

Zygomatic

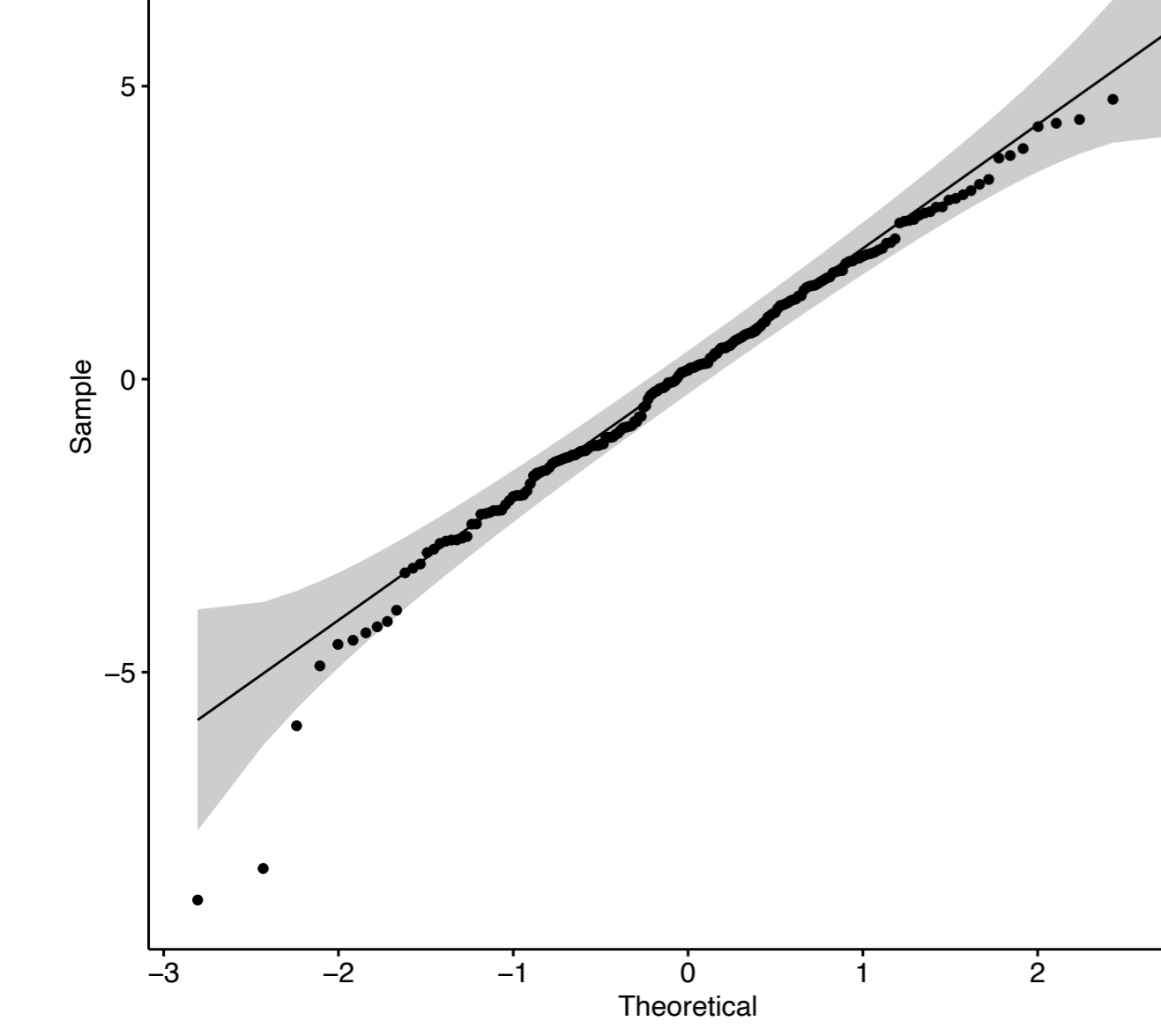

Supplement: Supplementary file 2 — Additional file 2. Morphological variable normal QQ plots for thirteen morphological traits used in this study. [file 12862_2024_2305_MOESM2_ESM.pdf]
